# Human non-CpG methylation patterns display both tissue-specific and inter-individual differences suggestive of underlying function

**DOI:** 10.1080/15592294.2021.1950990

**Published:** 2021-08-30

**Authors:** Philip Titcombe, Robert Murray, Matthew Hewitt, Elie Antoun, Cyrus Cooper, Hazel M Inskip, Joanna D Holbrook, Keith M Godfrey, Karen Lillycrop, Mark Hanson, Sheila J Barton

**Affiliations:** a MRC Lifecourse Epidemiology Unit, University of Southampton, Southampton, UK; b Institute of Developmental Sciences, University of Southampton, Southampton, UK; c Centre for Biological Sciences, University of Southampton, Southampton, UK; d NIHR Southampton Biomedical Research Centre, University of Southampton and University Hospital Southampton NHS Foundation Trust, Southampton, UK

**Keywords:** DNA methylation, non-CpG, methylation, CpG, CHH, CHG, CAT, CAC, CNN, hierarchical clustering analysis, HCA, cluster, tissue-specific, individual-specific, methylation patterns, human, umbilical cord, umbilical cord blood, muscle, peripheral blood, comparison

## Abstract

DNA methylation (DNAm) in mammals is mostly examined within the context of CpG dinucleotides. Non-CpG DNAm is also widespread across the human genome, but the functional relevance, tissue-specific disposition, and inter-individual variability has not been widely studied. Our aim was to examine non-CpG DNAm in the wider methylome across multiple tissues from the same individuals to better understand non-CpG DNAm distribution within different tissues and individuals and in relation to known genomic regulatory features.

DNA methylation in umbilical cord and cord blood at birth, and peripheral venous blood at age 12–13 y from 20 individuals from the Southampton Women’s Survey cohort was assessed by Agilent SureSelect methyl-seq. Hierarchical cluster analysis (HCA) was performed on CpG and non-CpG sites and stratified by specific cytosine environment. Analysis of tissue and inter-individual variation was then conducted in a second dataset of 12 samples: eight muscle tissues, and four aliquots of cord blood pooled from two individuals.

HCA using methylated non-CpG sites showed different clustering patterns specific to the three base-pair triplicate (CNN) sequence. Analysis of CAC sites with non-zero methylation showed that samples clustered first by tissue type, then by individual (as observed for CpG methylation), while analysis using non-zero methylation at CAT sites showed samples grouped predominantly by individual. These clustering patterns were validated in an independent dataset using cord blood and muscle tissue.

This research suggests that CAC methylation can have tissue-specific patterns, and that individual effects, either genetic or unmeasured environmental factors, can influence CAT methylation.

## Introduction

Epigenetics, the study of changes in gene expression that occur without alterations in the nucleotide sequence, plays a fundamental role in regulating the accessibility of DNA to the transcriptional machinery and the regulation of tissue-specific gene expression, as well as genomic imprinting and X chromosome inactivation in early development [1]. DNA methylation (DNAm) is a widely studied epigenetic modification and is normally examined in the context of a CG dinucleotide (CpG), where the cytosine base can be modified at the fifth carbon position by the addition of a methyl (CH_3_) group. DNAm in the CpG context has a well-established role in genomic regulation and control of gene expression, with evidence that altered CpG methylation may link early-life environmental exposures with later non-communicable diseases, such as cardiovascular disease or obesity [2,3]. Animal models have shown how different environmental factors, such as diet, exercise, and stress, can affect DNA methylation and gene expression [4–8].

DNA methylation outside the CpG context has been far less extensively studied, yet may be far more prevalent within the methylome; there are approximately 28 million CpG dinucleotides in the human genome but in excess of 556 million cytosines in a non-CpG context (USCS, hg19). For example, in human and mouse central nervous system neurons, ~2-6% of non-CpG sites are methylated, with a mode of ~20-25% methylation across those sites, whereas methylation levels at methylated CpG sites are typically ~60-90% [9,10]. However, CpG dinucleotides are relatively underrepresented in the genome [11], leading to far greater numbers of non-CpG sites, with non-CpG methylation representing up to half of all the methylation present; a study by Woodcock et al. suggests that, in DNA from human spleen, up to 54.5% of all methylation present are in a non-CpG context [12]. Levels of non-CpG methylation may depend highly on tissue type, with higher levels of specific non-CpG methylation reported in neurones and human embryonic stem cells, but present at much lower levels in other tissue types [9,10,13–15]. Studies in plants have found that non-CpG methylation predominantly occurs at base-pair triplicates [16,17], while studies in vertebrates examining non-CpG methylation have identified both symmetric CHG (H = A, C, or T) [18] and asymmetric CHH methylation patterns [14,19], at conserved positions in the genome [20,21], with the sequence flanking the cytosine position potentially modulating DNA methyltransferase 3A (DNMT3A)/DNMT3B binding, in conjunction with DNMT3L [22].

The role of non-CpG DNAm within the genome is unclear, and DNAm in different contexts may perform specific or overlapping functions. Non-CpG methylation has been linked to disease status such as in type 2 diabetes where increases in non-CpG methylation within the promoter of peroxisome proliferator-activated receptor-gamma coactivator (PGC-1α) were associated with impaired glucose tolerance. Moreover, PGC-1α non-CpG methylation was increased by free fatty acids, suggesting potential environmental modulation of the methylation status of these sites [23]. Non-CpG methylation has also been implicated in Alzheimer’s disease (AD), where non-CpG methylation patterns within the promoter of *Presenilin1* in human brain tissue were inversely correlated with expression in AD samples [24], suggesting active demethylation of non-CpG methylation, epigenetically regulating gene expression. Active demethylation of non-CpG methylation has been reported in other contexts [25] but remains understudied [26].

Differences in levels of non-CpG DNAm between human samples could be the result of stochastic change in the epigenome, but there are two main alternative explanations as to why non-CpG DNAm levels may differ in human samples. (1) Differences may exist between individuals: resulting from either environmental factors during development or an individual’s genetic sequence; (2) non-CpG DNAm patterns may differ by tissue type due to developmental programming during cell differentiation. If differences in non-CpG DNAm are not just purely due to random processes, then similarities in the patterns of non-CpG DNAm within tissue types or within individuals may be expected.

To investigate whether individual or tissue-specific factors may influence non-CpG methylation, here, we have examined both CpG and non-CpG methylation in placental cord and cord blood at birth, as well as peripheral venous blood collected at 12–13 y in a group of individuals (n = 20) from the Southampton Women’s Survey (SWS) cohort. Methylation data were captured using the Agilent SureSelectXT Human Methyl-Seq (SureSelect platform). Hierarchical clustering analysis (HCA) was applied to the CpG and non-CpG data to observe the clustering patterns. We then validated our findings in an independent dataset of 12 samples with two tissue types: cord blood and muscle.

## Methods

### Discovery data

In the discovery dataset, a total of 60 samples from the SWS cohort, a UK prospective cohort study in which women were recruited before the conception of the child [27], were interrogated by Agilent SureSelectXT Human Methyl-Seq capture and sequencing method (SureSelect). These 60 samples consisted of 20 individuals each analysed across three tissue types: cord blood, umbilical cord, and 12–13 y peripheral blood. Cord blood consists of B cells, granulocytes, monocytes, natural killer cells, nucleated red blood cells, and CD4 and CD8 T cells, and peripheral venous blood contains B cells, neutrophils, monocytes, natural killer cells, and CD4 and CD8 T cells. Individuals were selected on the basis of having DNA from all three tissues available in sufficient quantities (1 µg) and were split equally by sex (10 males/10 females), and five individuals from each quarter of the percent fat distribution from DXA measurements taken at age 8–9 y.

### Validation data

For the validation dataset, 12 samples were interrogated by Agilent SureSelect in total across five individuals. Muscle tissue samples from four males aged between 73 and 79 y from the Hertfordshire Sarcopenia Study (HSSe), a UK based cohort study [28], were assayed in duplicate (1A/B, 2A/B, 3A/B, 4A/B), and one cord blood sample (pooled from two individuals, both male) from the SWS was assayed as two duplicate pairs (5A/B and 5 C/D). Agreement in methylation levels between duplicates was assessed using a subset of 671,751 non-zero methylated non-CpG sites showing, on average, 93.4% of sites agreeing to within 10% methylation; reproducibility statistics and sample DNA quantities can be seen in Table S1.

### DNA extraction

DNA from muscle biopsy samples was stored and extracted as described previously [29]. For umbilical cord, a 5–10 cm segment was cut from the mid portion of each cord, immediately following delivery, flushed with saline to remove foetal blood, flash-frozen in liquid nitrogen, and stored at −80°C until required for DNA isolation. Peripheral blood was stored at −80°C until further processing. Genomic DNA from peripheral blood was extracted using QIAamp DNA Mini Kit (Qiagen, UK), following the manufacturers’ recommendations. Genomic DNA was prepared from umbilical cord, umbilical cord blood, and muscle tissue by a standard high salt method [30].

### Agilent sureselect methyl-seq data

Methyl-seq data were generated by the Centre for Genomic Research at the University of Liverpool using the Agilent SureSelect platform [31]. Both the discovery data and the validation data were cleaned, processed, and analysed using the same procedure detailed below. The data arrived as FASTQ files, trimming of adapters was performed using Cutadapt v1.2.1 with the option -O 3, so the 3' end of any reads which matched the adapter sequence for three base pairs or more were trimmed [32], and a minimum window quality score 20, using Sickle v1.2 [33]. Reads <10 bp were removed. The unmasked human genome was downloaded from UCSC, and the genome hash table was built using Extended Randomized Numerical Aligner (ERNE) Create [34]. The alignment against the genome was performed using ERNE-BS5 2 [35]. Unprocessed data contained paired-end reads and singleton reads. Singleton reads result from one read of a pair failing the Sickle quality control. The singlet files contained sequences whose pair had been removed due to poor sequence quality or adapter contamination. SureSelect data in the discovery dataset and the validation dataset produced similar summary statistics. Paired reads aligned uniquely to the genome at a greater rate (88.8% and 85.0%) than singleton reads (66.1% and 59.3%), and singleton reads were negligible in number (0.76 and 1.37 million reads) compared with paired-end reads (85.7 and 99.4 million reads) for discovery and validation datasets, respectively. As a result of this, singleton reads were not included in any analysis. For each sample, methylation calls (calculated by the number of methylated reads/total number of reads at each cytosine) were made using ERNE-METH 2 [35]. This provided the methylation level for each cytosine for each sample. Options ‘––annotations-bismark’ and ‘––annotations-erne’ were used during the methylation calling process to provide detailed cytosine context. Previous studies have demonstrated that reproducibility improvements are minimal beyond 30× read-depth [36]; therefore, a minimum read-depth of 30× was used for all downstream analyses. A flowchart summarizing the steps from SureSelect library preparation to statistical analysis is shown in Supplementary Figure S1.

### Statistical analysis

Data manipulation and summary statistics were created using Stata (version 15.0 and 16.0) and unix bash commands, and hierarchical cluster analysis was performed in R (version 3.5.1 and 3.6.1) using the ‘hclust’ command with complete linkage method and Euclidean distance as the metric to measure dissimilarity. Other linkage methods were also tested, with similar results (‘average linkage method’ and ‘weighted pair group method with arithmetic mean’). For hierarchical cluster analysis on non-CpG methylation, non-CpG sites were restricted to 671,751 sites where methylation was >0 for all 60 samples, i.e., all 20 individuals across all three tissue types had non-zero methylation values at these sites; these 671,751 non-CpG sites are frequently referred to as ‘non-zero’ methylation sites.

## Results

To understand tissue and individual differences in non-CpG methylation, DNA samples from 20 individuals in three tissue types (umbilical cord, cord blood, and peripheral blood) were interrogated for non-CpG (and CpG) methylation using Agilent SureSelect. Table 1 shows the number of sites for the CpG and non-CpG sites in the discovery dataset with over 30-fold read-depth, split by tissue type, and those sites covered in all 60 samples. In the discovery dataset, ~2.52 million CpG sites (>30× read-depth) were captured in at least one of the 60 samples, and similarly ~2.58 million in the validation dataset. When considering the number of CpG sites with over 30 reads across all 60 samples in the discovery dataset, the number reduced to 1,222,537 CpG sites. Over 17.6 million non-CpG sites (>30× read-depth) were captured in at least one of the 60 samples in the discovery dataset (and ~17.7 million in the validation dataset). The number of non-CpG sites with non-zero methylation in all of the 60 samples was 671,751, with a median methylation between 3.4% and 8.0% (median and 5th–95th percentile of methylation for each sample are shown in Table S2a). Of the 671,751 non-CpG sites that were non-zero methylated in the discovery dataset, 667,922 (99.4%) were covered with over 30 reads across all 12 samples in the validation dataset, and 586,435 of those were also non-zero methylated (Table S3).

**Table 1.** Summary of CpG and Non-CpG sites with over 30 reads by tissue type in discovery dataset. Summary tables with number of CpG and Non-CpG sites with over 30 reads by tissue type: **a)** in at least one individual; **b)** in all individuals; and **c)** having non-zero methylation in all individuals

**a)** Number of sites with over 30 reads in at least one individual	Cytosine Context	12–13 y peripheral blood	Cord blood	Umbilical cord	All 60 samples
	CpG	2,448,736	2,397,488	2,418,723	2,518,311
	Non-CpG	17,292,430	17,038,713	17,218,661	17,603,383
**b)** Number of sites with over 30 reads in *all* 20 individuals	Cytosine Context CpG	12–13 y peripheral blood 1,334,219	Cord blood 1,433,637	Umbilical cord 1,397,930	All 60 samples 1,222,537
	Non-CpG	10,843,753	11,321,097	10,940,859	9,779,206
**c)** Number of sites with over 30 reads and non-zero methylation values in all 20 individuals	Cytosine Context	12–13 y peripheral blood	Cord blood	Umbilical cord	All 60 samples
	CpG Non-CpG	1,026,360 1,657,153	1,098,718 1,923,066	1,075,501 2,633,428	862,472 671,751

Median methylation levels for the 671,751 non-CpG sites (identified in discovery dataset) were between 6.3% and 7.2% for samples in the validation data (Table S2(b) and Figure S2). The distribution of non-CpG and CpG methylation in relation to genomic features was examined in the validation dataset (Figure S3), finding a higher percentage of non-CpG sites vs. CpG sites located within introns (37.7% vs. 28.9%) and a lower percentage in promoters (30.1% vs. 38.4%). These differences were slightly larger when comparing CpG sites specifically to CAC or CAT sites that were non-zero methylated (Figure S3). Non-zero methylated CAC and CAT sites showed very similar distributions across genomic features (Figure S3), but median methylation levels for CAC sites were consistently higher than at CAT sites (Table S4). Promoter regions were defined as 2 kbp upstream and 500 bp downstream of transcriptional start sites.

### Discovery data: hierarchical cluster analysis of CpG and non-CpG methylation

**Figure 1.**
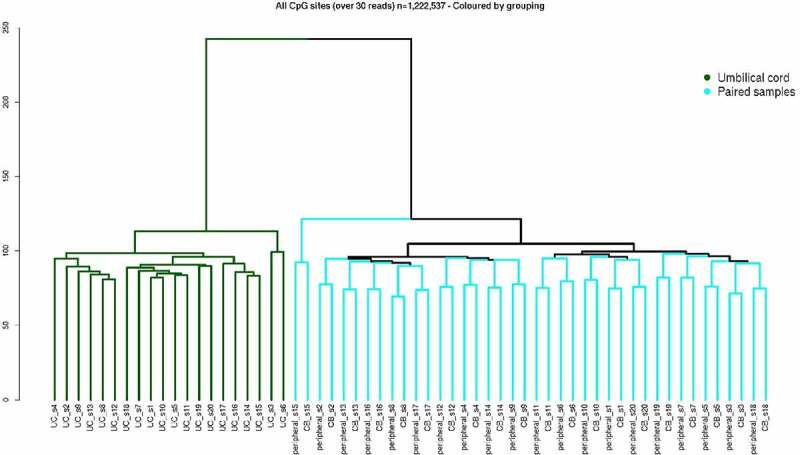
Hierarchical cluster analysis on CpG sites in discovery dataset. Analysis carried out on 1,222,537 CpG sites with >30× read-depth in each of the 60 samples. Cluster dendrograms shows separation of umbilical cord tissue (green), and remaining samples are grouped by pairs (cyan) of individuals’ cord blood (CB) and 12–13 y peripheral blood (peripheral) samples.

HCA was performed on DNA methylation patterns in umbilical cord, cord blood, and peripheral blood samples to investigate tissue-specific methylation patterns, and the relationship between inter- and intra-individual methylation. CpG methylation analysis was carried out on 1,222,537 sites, for which a minimum read-depth of 30-fold across all 60 samples was available (Figure 1). DNAm at CpG sites was found to separate first by tissue type, with cord blood and peripheral blood samples from the same individual clustering together, disparate from a cluster of umbilical cord samples.

To determine whether non-CpG methylated sites would cluster samples similarly to CpG sites, hierarchical cluster analysis was applied to non-CpG DNAm. Data were available for ~9.8 million non-CpG sites for which a minimum read-depth of 30-fold was met across all 60 samples. This data contained a large proportion of unmethylated sites, so the dataset was limited to ‘commonly methylated’ non-CpG sites that had greater than 30 reads and non-zero methylation levels across all three tissue types in the 20 individuals in the study, identifying 671,751 non-zero methylated non-CpG sites across all 60 samples for use in further analysis. Hierarchical clustering revealed differences in the way that samples were clustered: of the 60 samples, 10 samples clustered by tissue type – umbilical cord samples from 10 different individuals clustering together; 21 samples grouped by individual, with all 3 tissue samples (cord blood, umbilical cord, and peripheral blood) clustered together for 7 individuals; and 26 samples grouped into pairs of tissue, with cord blood and peripheral blood clustered together for 13 individuals – leaving 3 outlying samples (Figure S4).

### Sequence context of non-CpG methylated sites influences inter-tissue and inter-individual hierarchal clustering

**Figure 2.**
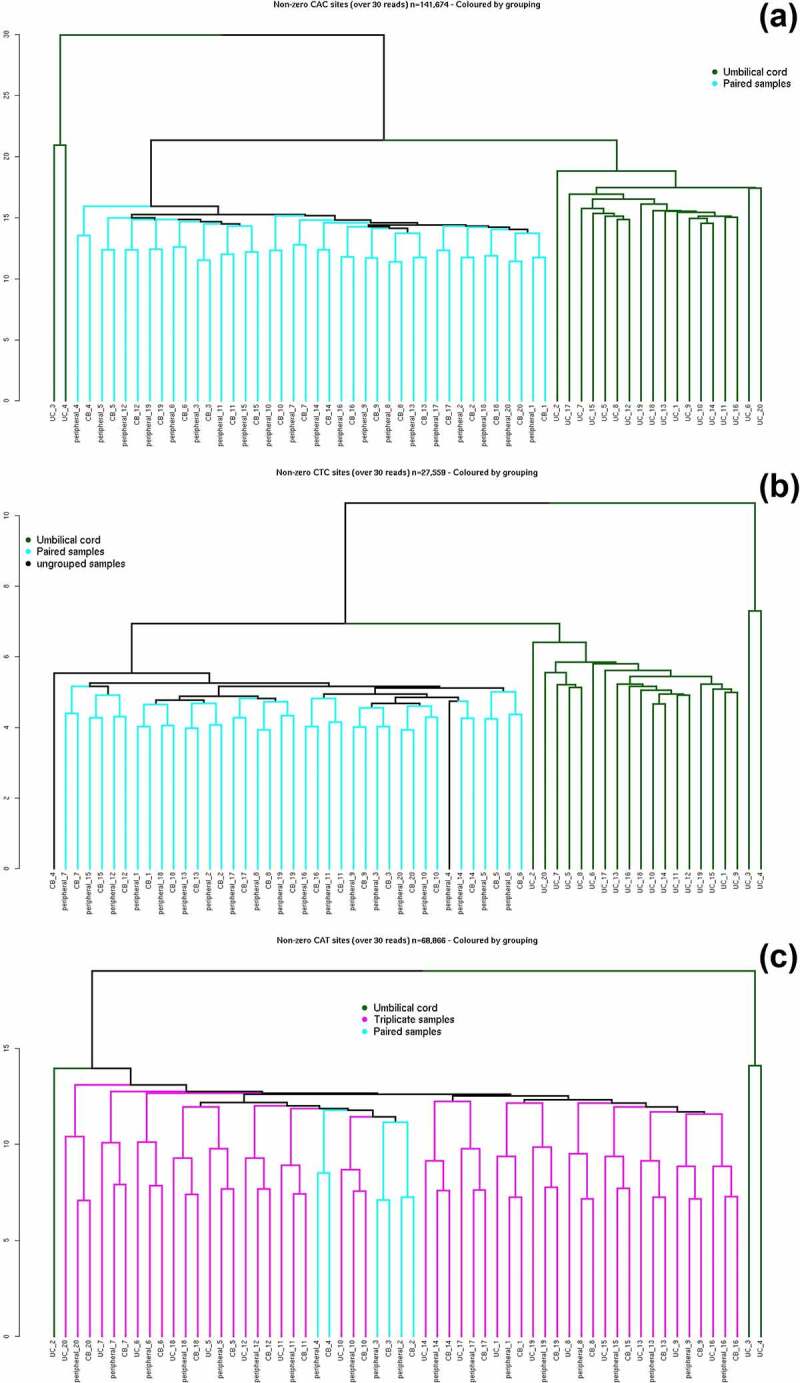
(a-c): Three dendrograms of non-CpG methylation sites in discovery dataset. Hierarchical cluster analysis was carried out using methylation sites with >30× read-depth across all 60 samples with at least one methylated read (non-zero methylated sites). Colour code: all three tissues from an individual clustered together in triplicates (magenta), pairs of peripheral blood and cord blood samples grouped by individual (cyan), umbilical cord samples not clustering by individual (green). UC = umbilical cord sample, CB = cord blood sample, peripheral = 12–13 y peripheral blood. Restricted to three separate cytosine sequence contexts: (a) CAC sites (n = 141,674), (b) CTC sites (n = 27,559), and (c) CAT sites (n = 68,866).

Analysing all non-zero non-CpG sites together combines cytosines from a range of different underlying sequence contexts, which may obscure specific patterns in their DNA methylation profiles, and it has been previously suggested that the cytosine sequence context (CHG and CHH, in the 5' to 3' direction, where H = A, C, or T) may have an influence on methylation patterns in mammals [37]. Non-CpG methylation was, therefore, separated into 12 different cytosine contexts and analysed separately: CTG, CAG, CCG, CTT, CAT, CCT, CTA, CAA, CCA, CTC, CAC, and CCC (Table S3). Hierarchical cluster analysis revealed clear differences in clustering patterns depending on the adjacent DNA sequence of the non-CpG sites. Dendrograms for non-zero methylated CAC (n = 141,674), CTC (n = 27,559), and CAT (n = 68,866) sites are shown in Figure 2(a,b,c). For the other nine non-CpG cytosine specific contexts, samples still showed some clustering by tissue or individual but displayed less distinct clustering patterns and are shown in Figure S5(a-i).

Using DNAm values from cytosines in the CAC or CTC context, samples clustered by tissue type with DNA samples from cord blood and peripheral blood clustering together in each individual, but separately from umbilical cord tissue (Figure 2(a,b)). Samples were then paired by individuals within the cluster of peripheral and cord blood, with cytosines in the CAC context pairing all 20 individuals, and 19 of 20 individuals pairing using CTC sites. In cluster analysis using DNAm occurring at CAT sites, samples grouped predominately by individual, with DNA samples from cord tissue, cord blood, and peripheral blood forming triads by individuals (Figure 2c). Non-zero methylated CAT sites showed a different pattern of clustering (separation by individuals) compared with using non-zero methylation data exclusively from CAC or CTC sites (tissue separation).

Given these observations that clustering patterns are affected by different cytosine contexts of non-CpG DNAm, we next examined different cytosine contexts for CpG methylation to determine whether clustering of samples varied between CGA, CGC, CGG, and CGT methylation. Differences were seen between the four analyses, whereby CGT and CGG methylation sites were able to separate out first by umbilical cord tissue, and then successfully cluster all remaining peripheral and cord blood samples by pairing individuals. CGC and CGA sites were similar to CGT and CGG sites; but within the 40 samples of peripheral and cord blood, not all samples were clustered by individuals (Figure S6(a-d)).

### Different cytosine contexts in non-CpG methylation cluster analysis – validation data

**Figure 3.**
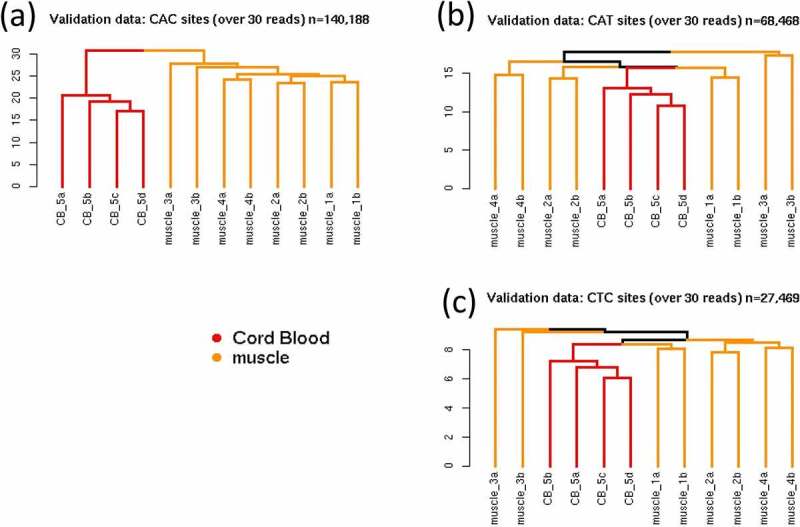
(a-c): Dendrogram of non-CpG methylation in validation dataset. Muscle tissue samples (yellow) were assayed in four individuals in duplicate (1A/B, 2A/B, 3A/B, 4A/B), and one cord blood sample (red) from the SWS was assayed in quadruplicate (5A/B/C/D) (pooled from two individuals). Hierarchical cluster analysis was carried out on samples from validation dataset using non-CpG sites (>30× read-depth) overlapping with 671,751 non-zero non-CpG sites from discovery dataset, restricted to (a) 140,188 CAC sites, (b) 68,468 CAT sites, and (c) 27,469 CTC sites.

Having seen that using non-zero non-CpG methylation sites could generate different clustering patterns depending on the genomic sequence adjacent to the cytosines, we wanted to examine whether this phenomenon could be validated in an independent dataset. In order to test whether this pattern would occur not only in a different set of individuals but also in a tissue type not analysed in the discovery data, HCA was conducted in a dataset of 12 samples consisting of four individuals in duplicate using muscle tissue, and cord blood data from a pooled DNA sample carried out in two duplicate pairs. Dendrograms were created using the subset of 671,751 non-CpG sites that were non-zero methylated in the discovery dataset, provided these sites had over 30 reads in the validation dataset. Figure 3 shows dendrograms for three different cytosine contexts: CAC, CTC, and CAT. This shows that methylation at these CAC sites clustered samples by tissue type first, then individuals within muscle tissue (as with the discovery dataset), whereas methylation at these CAT sites clustered samples by individuals, with no initial separation of muscle from cord blood samples.

## Discussion

Little is known to date on the functional significance of non-CpG methylation. In this study, we examined non-CpG DNAm across multiple tissues from the same individuals to better understand differences in tissue specificity and inter-individual variability of non-CpG methylation. We found that hierarchical cluster analysis, using DNAm data from non-CpG sites in cord blood, peripheral blood, and umbilical cord, clustered samples by individual and/or separated certain tissue types. If measured non-CpG DNAm were purely the result of randomness in the epigenome, the expected result from our hierarchical clustering would be samples clustering at random or not at all. This demonstrates that non-CpG methylation is not just occurring randomly in the genome but that non-CpG methylation patterns can differ by tissue type and that these differences may in part be driven by an individual’s genomic or environmental exposures.

In addition to this, non-CpG methylation in certain genomic contexts (e.g., CAC) separated samples by tissue type, grouping samples from different individuals into an umbilical cord cluster, and then grouping cord blood and peripheral blood together from each individual. A similar pattern was observed when analysing CpG sites. However, using non-zero DNAm at CAT sites, predominately all three samples from an individual clustered together (17 of 20 individuals clustered in their triplicates) rather than separating by tissue type, suggesting that some non-CpG DNAm sites are more tissue-specific and others more susceptible to individual effects. Using the subset of methylated non-CpG sites identified from the discovery analysis phase, the concept of cytosine sequence context driving tissue- or individual-based clustering (for CAC and CAT, respectively) was validated in an independent dataset using cord blood and muscle tissue – a tissue type that had not been used in the discovery data. This suggests that the subset of non-zero methylated non-CpG DNAm sites identified here may have relevance across several tissue types and a broad spectrum of people.

The distribution of non-CpG and CpG sites differed in relation to genomic features, especially within intronic and promoter regions. It is also worth noting that even though non-zero methylated CAC and CAT sites clustered samples differently (by tissue or by individual, respectively), these sites show very similar distributions across genomic features. This suggests that the differences in tissue/individual clustering patterns using methylated CAC and CAT sites may be due to varying levels of methylation across these sites rather than their distribution in relation to genomic features. Where samples cluster separately by tissue type using non-zero methylated CAC sites, cord and peripheral blood were found to cluster together, indicating a similar methylation profile within these tissue types. This is suggestive of lineage-specific non-CpG methylation patterns that have potentially been maintained from a common precursor cell type.

Existing literature on non-CpG methylation is very limited, focusing mainly on stem cells and brain tissue. In addition, studies on non-CpG methylation are mostly limited to two base-pair sequence context in the 5' to 3' direction (CpA, CpC, or CpT) [38–40]. Here, we present evidence that a three base-pair cytosine sequence context can either display tissue-specific methylation (CAC) or individual-specific methylation patterns (CAT) or show no clear clustering by tissue or individual (CAA and CAG). This suggests that restricting analysis of non-CpG methylation data to a two base-pair context may be grouping together disparate methylation patterns (e.g., CpA = CAT, CAC, CAA and CAG) and, therefore, concealing important differences connected to the third base in the triplicate.

CpG sites are symmetrical, whereby there is a cytosine and guanine on the complementary strand in the 3' to 5' direction and, if methylated, CpG methylation generally occurs on both strands (reciprocal methylation). As a result, CpG methylation can be maintained during cell replication by DNA methyltransferase 1 (DNMT1) [41]. However, it has been shown that some CpG sites are hemi-methylated and that CpG hemi-methylation can be inherited over several cell divisions, suggesting that, although most hemi-methylated CpG sites become fully methylated during cell divisions, hemi-methylation in some CpG sites may be a stable epigenetic state [42]. CHH sites, such as CAC, are not symmetrical and so any methylation occurring at CHH sites is also hemi-methylated. As samples in our study maintained a tissue/individual specific signature using only subgroups of CHH methylation, this suggests that there may exist some form of active maintenance of methylation for non-symmetrical non-CpG sites too.

In the discovery data, samples 3 and 4 from umbilical cord clustered separately from all other samples when using non-CpG data. These samples displayed noticeably higher non-CpG methylation values than any other samples, but the reason for such deviation is not known. Interestingly, these individuals did not cluster separately when using CpG data or when using non-CpG data from cord blood or peripheral blood; it is only non-CpG sites from umbilical cord samples for these individuals that differed in methylation. Umbilical cord is a heterogeneous mixture of tissues types [43], so it is possible that more of a particular tissue type that contains higher levels of non-CpG methylation was present in the aliquot of umbilical cord tissue used for these two individuals. Another explanation could be potential unknown environmental factors, but this would imply that those factors only affected non-CpG DNAm specifically in umbilical cord tissue samples and not any other measured methylation.

One of the strengths of this study is the increased coverage of the methylome provided by Agilent SureSelect data compared with more widely used methods, such as the Infinium 850 K EPIC array, which only covers ~850,000 methylation sites and is focused on CpG sites. Our SureSelect Methyl-seq dataset contains methylation data on ~2.52 million CpG sites (>30× read-depth) or 1,222,537 CpG sites when selecting CpG sites with over 30 reads across all 60 samples in the discovery dataset; coverage of non-CpG sites was ~17.6 million (>30× read-depth) or 671,751 sites when selecting non-zero percent methylated sites with over 30 reads across all 60 samples in the discovery dataset.

A further strength of this study is the multiple different tissue types for each individual, thus allowing for comparisons across tissue types and individuals. Having access to two independent cohorts with SureSelect data on CpG and non-CpG data was also advantageous and made it possible for us to validate our findings from the discovery data. One more novel aspect of this study is the tissue types used, which are not commonly examined for their non-CpG methylation status: cord blood, umbilical cord, muscle, and peripheral blood samples – tissue samples that are generally quite accessible to researchers.

The examination of CGA, CGC, CGG, and CGT sites suggests that, in contrast to observations of cytosine contexts of non-CpG sites, the cytosine context of CpG sites may have less of an effect on the methylation values and that the clear differences between tissue- and individual-driven clustering seen in cytosine sequence contexts may be unique to non-CpG methylation.

One of the limitations of this study is the sequence-based nature of the SureSelect assay, meaning that in a separate SureSelect assay, not all the sites identified in this study will be guaranteed to meet the minimum read-depth cut-off of >30-fold that we used. This would make it difficult for other researchers to replicate our observations using exactly the same non-CpG sites as us. However, we saw 99.4% of non-zero methylated non-CpG sites in our discovery dataset in our validation dataset (with over 30-reads). Even if the subset of non-CpG sites identified by other researchers does not overlap exactly with those used in this study, one option could be to use a subset of the non-CpG sites identified here. In the analysis here, we have presented data in relation to non-CpG methylation in the context of trinucleotides (CHG and CHH sites) as non-CpG methylation in this context has been the most widely reported [14,19–21]; there is some evidence that suggests additional nucleotides outside of CHG and CHH sites may also play a role in determining methylation [14,22], but this was outside the scope of our study.

In terms of measurement error on SureSelect platform, Teh et al. [36] have previously shown that using a 30× read-depth coverage and 1 µg of DNA, 71% of probes agreed to within an absolute difference of 5% methylation with the replicate sample, and this increased to 91% agreeing within 10% methylation. We see a very similar level of agreement at methylated non-CpG sites (validation data shown in Table S1) with an overall average of 93.4% of data agreeing to within 10% methylation. Median methylation levels for the 671,751 non-CpG sites (identified in discovery dataset) are between 6.3% and 7.2% in the validation data, and measurement error on the array is not negligible compared with this. However, despite the impact of possible measurement error from the array and relatively low levels of methylation across non-CpG DNAm sites compared with CpG sites, we still saw our data clustering in meaningful ways when restricted to only non-zero methylated non-CpG sites.

The process outlined in this paper identified a subset of non-CpG sites that are commonly methylated across 20 individuals and in each of their three tissue types. The results were validated in an independent dataset, including the use of previously unused tissue types; this suggests that there may exist a subset of non-CpG sites that are commonly methylated within the population and also across multiple tissue types. Therefore, similar to our approach of using previously untested tissue type in our validation data, other researchers may be able to examine these same non-CpG sites without necessarily having similar cohort or similar tissue types to those seen in this study.

Although the functionality of non-CpG methylation has not been comprehensively explained in mammals, it is clear that non-CpG methylation profiles can be used to differentiate between tissue types and between individuals. In addition, certain subsets of non-CpG methylation sites are better able to differentiate between tissue types, while others are able to more easily differentiate between individuals. More research is needed to gain insight as to why data from some non-CpG contexts cluster by individuals and others principally by tissue type and also what functional significance these, or any other, non-CpG sites may have in the development of health and disease.

## Supplementary Material

Supplemental Material
