# Supplementary material for: Human non-CpG methylation patterns display both tissue-specific and inter-individual differences suggestive of underlying function
Source: Epigenetics. 2021 Aug 30;17(6):653–64. doi: 10.1080/15592294.2021.1950990 (PMC9235887; doi:10.1080/15592294.2021.1950990)
Supplement: Supplemental Material [file KEPI_A_1950990_SM0731.zip › supplementary/Supplementary tables and figures_revised_with_descriptions.docx]

Supplementary tables and figures

**Supplementary Table S1:**

| Samples | % within 10% | % within 5% | DNA quantity |
| --- | --- | --- | --- |
| \|1a - 1b\| | 93.0 | 68.1 | 760ng |
| \|2a - 2b\| | 93.3 | 68.6 | 1µg |
| \|3a - 3b\| | 88.8 | 61.2 | 663ng |
| \|4a - 4b\| | 92.6 | 67.6 | 1µg |
| \|5a - 5b\| | 95.1 | 72.2 | 1µg |
| \|5c - 5d\| | 97.7 | 79.8 | 3µg |
| Average | 93.4 | 69.6 |  |

**Table S1: Absolute methylation difference between duplicates and DNA quantities in validation dataset:** Using 671,751 non-zero methylated non-CpG sites identified in the discovery data, and examining the agreement between replicate samples in the validation data. Values shown here represent the % of non-CpG sites where the absolute value of the difference in methylation between replicates is less than 5% and 10% methylation, and an overall average. DNA quantities used for each sample is also shown by duplicate pair.

**Supplementary Table S2a:**

| Individual | 12-13yr Peripheral Blood | | Umbilical Cord Blood | | Umbilical Cord | |
| --- | --- | --- | --- | --- | --- | --- |
|  | Median | 5th-95th percentile | Median | 5th-95th percentile | Median | 5th-95th percentile |
| 1 | 3.4 | (1.0, 8.0) | 3.5 | (1.0, 8.3) | 4.3 | (1.4, 10.0) |
| 2 | 3.4 | (1.0, 8.0) | 3.5 | (1.0, 8.3) | 5.4 | (1.7, 12.2) |
| 3 | 3.5 | (1.0, 8.2) | 3.5 | (1.1, 8.0) | 8.0 | (3.1, 16.6) |
| 4 | 3.5 | (0.9, 8.5) | 4.5 | (1.5, 9.9) | 7.7 | (3.0, 16.0) |
| 5 | 3.4 | (0.9, 8.7) | 3.4 | (1.0, 8.0) | 4.5 | (1.6, 10.2) |
| 6 | 3.5 | (0.9, 8.7) | 3.4 | (0.9, 8.3) | 4.5 | (1.2, 11.0) |
| 7 | 3.4 | (0.9, 8.7) | 3.4 | (0.9, 8.3) | 4.6 | (1.5, 10.7) |
| 8 | 3.8 | (1.3, 8.5) | 3.4 | (1.0, 7.9) | 4.7 | (1.7, 10.4) |
| 9 | 3.4 | (1.0, 8.1) | 3.4 | (1.0, 8.1) | 3.9 | (1.2, 9.3) |
| 10 | 3.4 | (0.9, 8.5) | 3.4 | (0.9, 8.1) | 3.6 | (1.1, 8.6) |
| 11 | 3.4 | (0.9, 8.4) | 3.5 | (1.0, 8.1) | 3.8 | (1.1, 9.2) |
| 12 | 3.4 | (0.9, 8.5) | 3.8 | (1.2, 8.6) | 4.2 | (1.4, 9.7) |
| 13 | 3.4 | (1.0, 7.9) | 3.4 | (1.0, 8.3) | 3.8 | (1.0, 9.5) |
| 14 | 3.5 | (1.0, 8.4) | 3.5 | (1.0, 8.5) | 3.9 | (1.1, 9.3) |
| 15 | 3.6 | (1.0, 8.6) | 3.7 | (1.2, 8.5) | 4.3 | (1.5, 10.0) |
| 16 | 3.5 | (1.0, 8.1) | 3.5 | (1.0, 8.3) | 3.9 | (1.1, 9.4) |
| 17 | 4.1 | (1.3, 9.0) | 3.5 | (1.0, 8.3) | 3.8 | (1.0, 10.2) |
| 18 | 3.4 | (1.0, 8.3) | 3.6 | (1.1, 8.2) | 3.9 | (1.1, 9.6) |
| 19 | 3.4 | (0.9, 8.5) | 3.6 | (1.0, 8.3) | 3.9 | (1.0, 10.0) |
| 20 | 3.4 | (1.0, 8.1) | 3.5 | (1.1, 8.1) | 4.6 | (1.4, 11.1) |

**Supplementary Table S2b:**

| Sample | Tissue type | n | Median | 5^th^-95^th^ percentile |
| --- | --- | --- | --- | --- |
| 1a | Muscle | 671,668 | 6.7 | (1.7, 16.1) |
| 1b | Muscle | 671,235 | 6.7 | (1.6, 16.2) |
| 2a | Muscle | 671,700 | 6.8 | (2.0, 15.9) |
| 2b | Muscle | 671,139 | 6.8 | (1.5, 16.7) |
| 3a | Muscle | 669,538 | 6.8 | (1.0, 17.9) |
| 3b | Muscle | 671,340 | 7.2 | (1.5, 18.0) |
| 4a | Muscle | 671,475 | 6.7 | (1.6, 16.3) |
| 4b | Muscle | 670,982 | 6.7 | (1.5, 16.5) |
| 5a | Cord Blood | 671,694 | 6.3 | (1.7, 14.4) |
| 5b | Cord Blood | 671,675 | 6.5 | (1.8, 14.5) |
| 5c | Cord Blood | 671,750 | 6.5 | (2.6, 13.1) |
| 5d | Cord Blood | 671,731 | 6.7 | (2.5, 14.0) |

**Table S2 (a-b): Methylation summary statistics for subset of 671,751 non-zero methylated non-CpG sites**: (**a)** Discovery dataset: 671,751 non-zero non-CpG sites (with over 30x read-depth), statistics shown are: median and 5-95^th^ percentiles of methylation (%) at these non-CpG sites broken down by three tissue types used. **(b)** Validation dataset: n describes the number of non-missing sites in validation dataset (with over 30x read-depth) overlapping with 671,751 sites identified in the discovery dataset. Statistics shown are: median, 5-95^th^ percentiles of methylation, and tissue type used.

**Supplementary Table S3:**

| Cytosine context | Discovery data (>30 reads) | Validation data (>30 reads) | |
| --- | --- | --- | --- |
|  | #sites non-zero methylated | #sites overlap (%) | #sites non-zero overlap (%) |
| CAA | 90,912 | 90,448 (99.5%) | 79,001 (86.9%) |
| CAC | 141,674 | 140,188 (99.0%) | 127,764 (90.2%) |
| CAG | 141,517 | 140,790 (99.5%) | 126,165 (89.2%) |
| CAT | 68,866 | 68,468 (99.4%) | 59,782 (86.8%) |
| CCA | 37,993 | 37,906 (99.8%) | 31,818 (83.7%) |
| CCC | 18,593 | 18,547 (99.8%) | 15,675 (84.3%) |
| CCG | 25,025 | 24,795 (99.1%) | 22,514 (90.0%) |
| CCT | 29,168 | 29,116 (99.8%) | 24,311 (83.3%) |
| CTA | 16,560 | 16,521 (99.8%) | 13,549 (81.8%) |
| CTC | 27,559 | 27,469 (99.7%) | 23,304 (84.6%) |
| CTG | 42,342 | 42,233 (99.7%) | 36,344 (85.8%) |
| CTT | 31,542 | 31,441 (99.7%) | 26,208 (83.1%) |

**Table S3: Breakdown of non-zero methylated non-CpG sites:** number of non-CpG sites across 12 different cytosine contexts in discovery, n (%) of sites overlapping with over 30 reads in all 12 samples from validation data, and overlap of sites with over 30 reads and non-zero methylated n (%) in all 12 samples from validation data.

**Supplementary Table S4:**

| Individual | Median (5th - 95th percentile) methylation in 12-13 year Peripheral blood | | Median (5th - 95th percentile) methylation in Cord blood | | Median (5th - 95th percentile) methylation Umbilical cord | |
| --- | --- | --- | --- | --- | --- | --- |
|  | CAC | CAT | CAC | CAT | CAC | CAT |
| 1 | 4.1 (1.3, 8.9) | 3.5 (1.0, 7.9) | 4.2 (1.3, 9.3) | 3.5 (1.0, 8.1) | 5.4 (1.9, 12.1) | 4.5 (1.5, 9.6) |
| 2 | 4.1 (1.3, 8.9) | 3.5 (1.1, 7.8) | 4.2 (1.3, 9.3) | 3.6 (1.0, 8.2) | 6.6 (2.3, 14.3) | 5.5 (1.8, 11.8) |
| 3 | 4.2 (1.3, 9.2) | 3.5 (1.0, 8.0) | 4.2 (1.4, 8.8) | 3.6 (1.2, 7.8) | 9.6 (4.0, 18.8) | 8.1 (3.2, 16.1) |
| 4 | 4.2 (1.2, 9.6) | 3.6 (1.0, 8.3) | 5.4 (1.9, 10.9) | 4.6 (1.5, 9.6) | 9.3 (3.9, 18.3) | 7.9 (3.2, 15.4) |
| 5 | 4.1 (1.1, 9.6) | 3.4 (0.9, 8.6) | 4.1 (1.3, 8.9) | 3.4 (1.0, 7.8) | 5.6 (2.1, 12.3) | 4.6 (1.7, 9.7) |
| 6 | 4.2 (1.2, 9.7) | 3.6 (1.0, 8.5) | 4.1 (1.2, 9.3) | 3.4 (1.0, 8.2) | 5.6 (1.7, 13.3) | 4.5 (1.3, 10.5) |
| 7 | 4.2 (1.1, 9.7) | 3.5 (0.9, 8.5) | 4.1 (1.1, 9.2) | 3.4 (0.9, 8.2) | 5.8 (2.0, 12.8) | 4.8 (1.6, 10.2) |
| 8 | 4.6 (1.7, 9.2) | 3.9 (1.3, 8.3) | 4.1 (1.3, 8.8) | 3.5 (1.0, 7.7) | 5.8 (2.2, 12.5) | 4.8 (1.8, 9.8) |
| 9 | 4.1 (1.3, 9.2) | 3.5 (1.0, 7.9) | 4.2 (1.3, 9.2) | 3.5 (1.0, 7.9) | 4.9 (1.6, 11.2) | 4 (1.3, 8.9) |
| 10 | 4.1 (1.2, 9.6) | 3.5 (1.0, 8.3) | 4.1 (1.2, 9.0) | 3.4 (1.0, 8.0) | 4.6 (1.6, 10.8) | 3.7 (1.2, 8.2) |
| 11 | 4.1 (1.2, 9.4) | 3.5 (0.9, 8.3) | 4.2 (1.3, 9.0) | 3.5 (1.1, 7.9) | 4.8 (1.5, 11.2) | 3.9 (1.2, 8.9) |
| 12 | 4.1 (1.1, 9.5) | 3.5 (0.9, 8.3) | 4.5 (1.5, 9.5) | 3.8 (1.2, 8.3) | 5.3 (1.9, 11.9) | 4.3 (1.5, 9.2) |
| 13 | 4.1 (1.3, 8.8) | 3.4 (1.0, 7.7) | 4.2 (1.2, 9.4) | 3.5 (1.0, 8.1) | 4.9 (1.4, 11.8) | 3.9 (1.1, 9.2) |
| 14 | 4.3 (1.3, 9.6) | 3.6 (1.0, 8.2) | 4.2 (1.2, 9.7) | 3.5 (1.0, 8.2) | 4.9 (1.6, 11.3) | 4 (1.2, 8.9) |
| 15 | 4.3 (1.3, 9.6) | 3.6 (1.0, 8.4) | 4.5 (1.5, 9.4) | 3.8 (1.2, 8.3) | 5.5 (2.0, 12.5) | 4.4 (1.5, 9.5) |
| 16 | 4.2 (1.4, 9.0) | 3.6 (1.1, 8.0) | 4.3 (1.3, 9.4) | 3.6 (1.0, 8.1) | 4.9 (1.6, 11.4) | 4 (1.2, 9.0) |
| 17 | 4.9 (1.7, 10.0) | 4.1 (1.4, 8.7) | 4.2 (1.3, 9.3) | 3.6 (1.0, 8.2) | 4.9 (1.3, 12.6) | 3.9 (1.0, 9.9) |
| 18 | 4.1 (1.2, 9.2) | 3.5 (1.0, 8.1) | 4.2 (1.4, 9.0) | 3.6 (1.1, 8.1) | 4.9 (1.5, 11.9) | 4 (1.2, 9.2) |
| 19 | 4.1 (1.1, 9.4) | 3.5 (1.0, 8.4) | 4.3 (1.3, 9.2) | 3.6 (1.0, 8.3) | 5 (1.4, 12.4) | 4 (1.1, 9.7) |
| 20 | 4.2 (1.3, 9.1) | 3.5 (1.1, 7.9) | 4.3 (1.4, 9.0) | 3.6 (1.1, 7.9) | 5.8 (1.9, 13.8) | 4.7 (1.5, 11.0) |

**Table S4: Percentage methylation for non-zero CAC and CAT sites in discovery dataset:** Median and (5^th^-95^th^ percentile) %methylation for non-zero CAC (n=141,764) and non-zero CAT methylation (n=68,866) in discovery dataset (60 samples). For each sample comparison, median methylation was consistently higher at CAC sites compared to CAT sites; the difference averaged +0.6% to +0.8% for peripheral blood and cord blood, and +0.9% to +1.5% for umbilical cord.

**Supplementary Figure S1:**

**
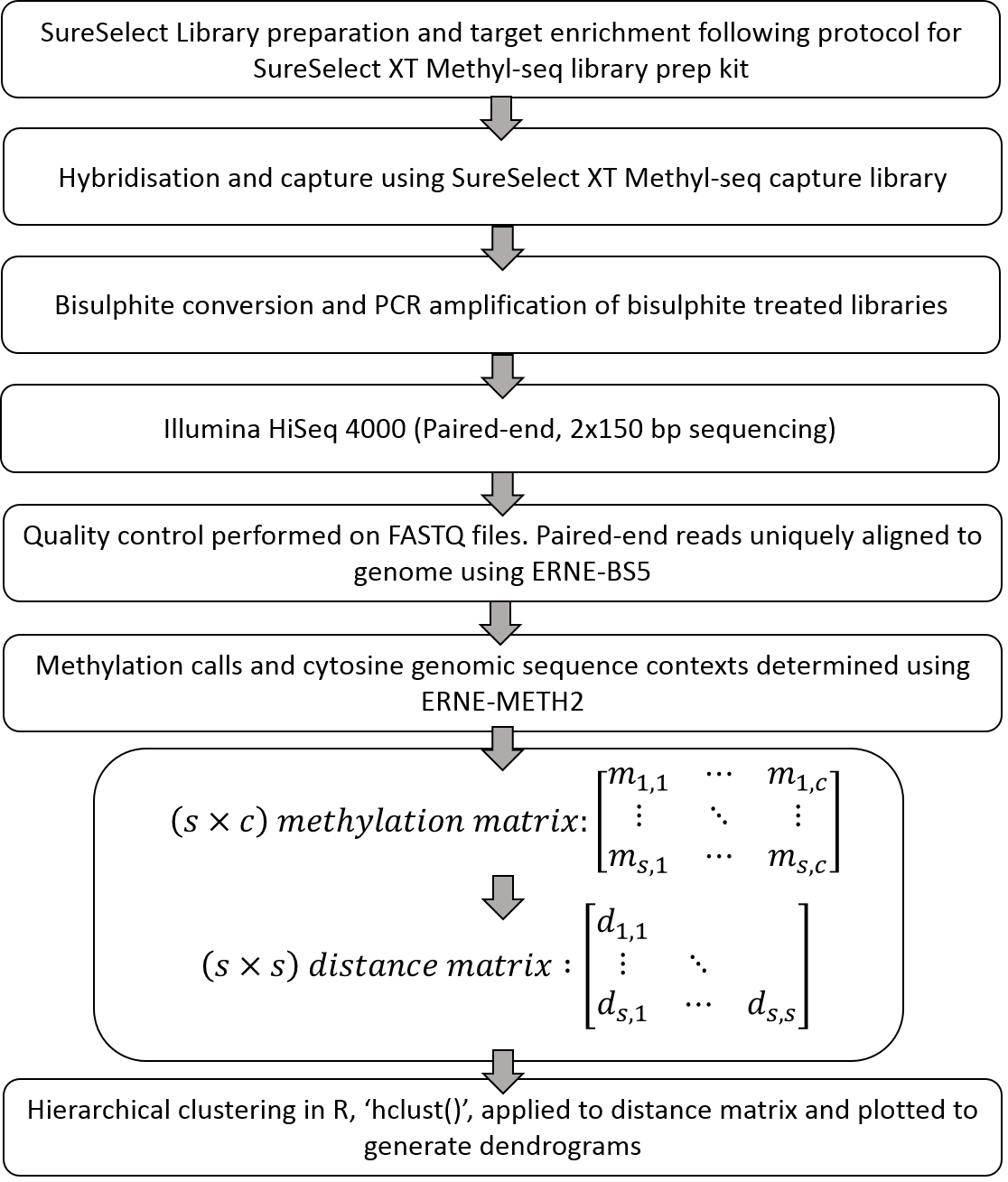
**

**Figure S1: SureSelect library preparation to hierarchical cluster analysis flowchart**: Matrices show a generalised example of an (s x c) methylation matrix, where s= number of samples in data set (in discovery data s=60, in validation data s=12), and c=number of cytosines (e.g. for non-zero methylated cytosines in a CAT context, c=68,866). An (s x s) Euclidian distance matrix was calculated by applying ‘dist()’ command to the methylation matrix in R.

**Supplementary Figure S2:**


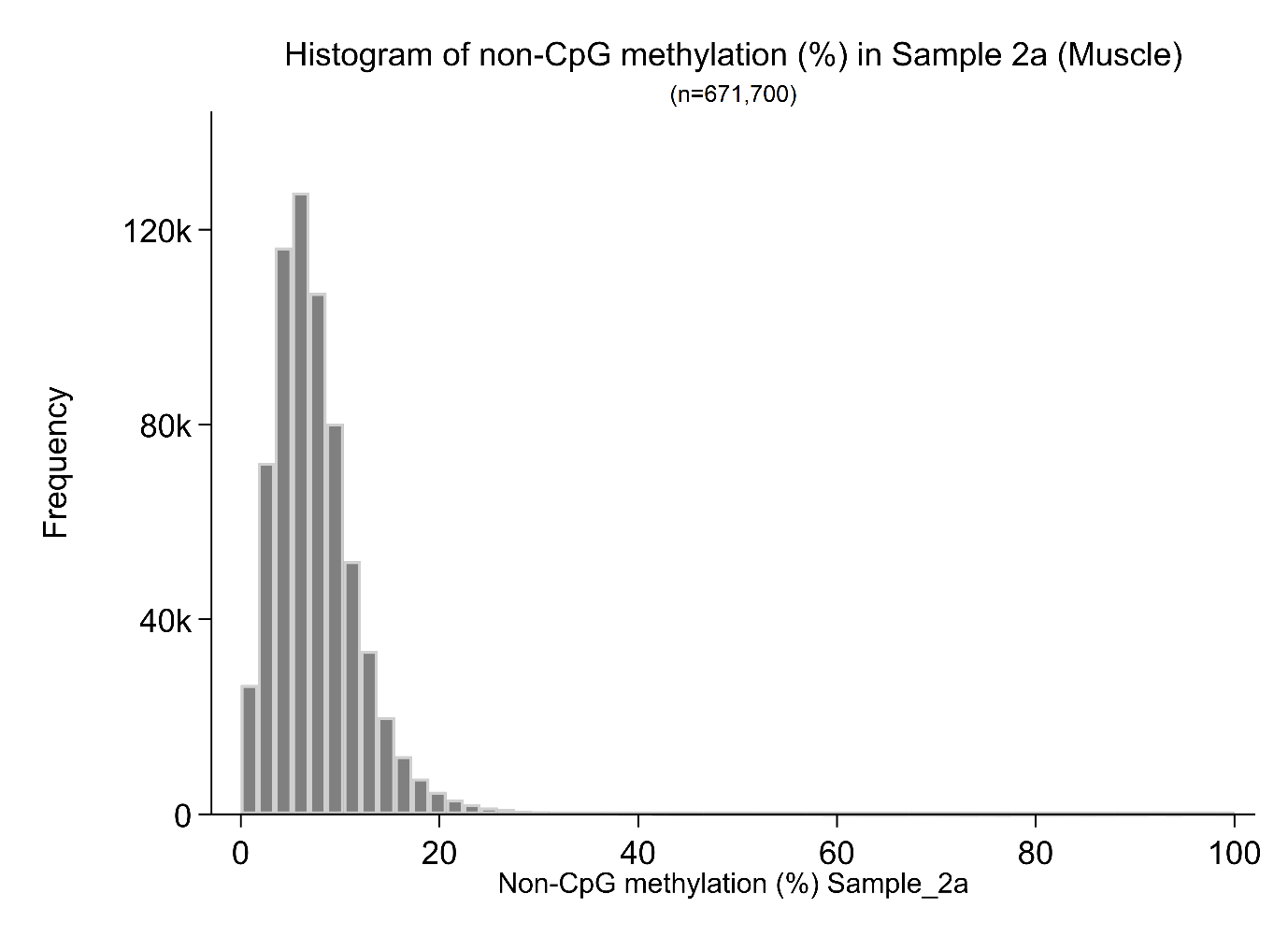


**Figure S2: Histogram of methylation at selected non-CpG sites in muscle:** Histogram of methylation in Sample 2a (muscle, 1µg) from validation dataset (n=671,700), using non-CpG sites (over 30x read-depth) overlapping with subset of 671,751 non-zero methylated non-CpG sites identified from discovery data.

**Supplementary Figure S3:**

**
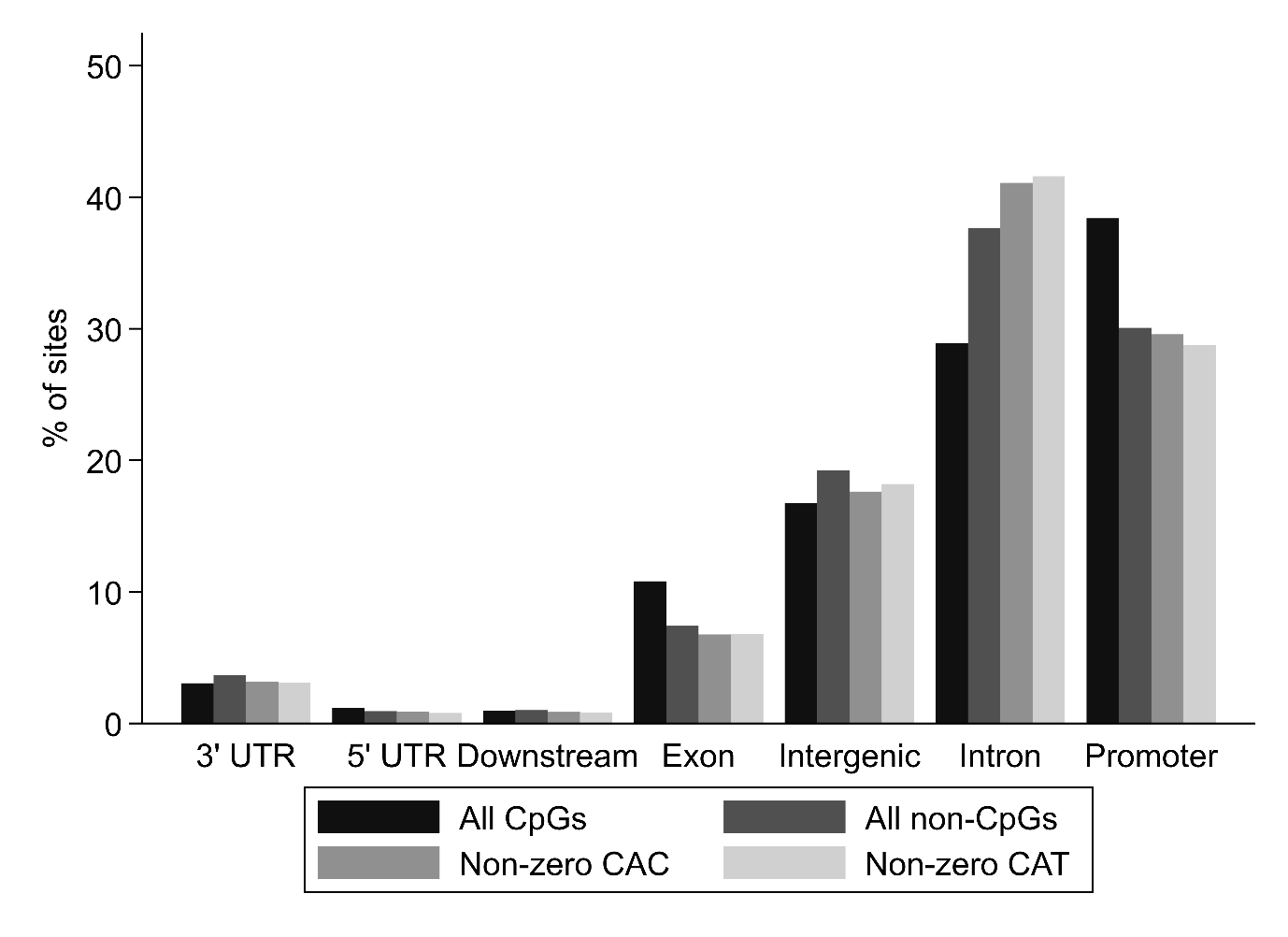
**

**Figure S3: Relation of CpG and non-CpG methylation to genomic features:** Breakdown of sites location in relation to genomic features in the validation data using: all CpG sites (n=2,584,074) and non-CpG sites (n=17,714,342) on SureSelect platform with >30x read-depth in validation data, and non-zero methylated CAC (n=141,634) and CAT (n=68,808) sites within the 671,751 non-zero non-CpG sites identified from the discovery data. The annotations for genomic regions in this work were sourced from the UCSC hg19 genome assembly based on the knownGene track and applied using the ChIPseeker package in R. Promoter: a region 2kbp upstream and 500bp downstream of transcriptional start sites. Downstream: a region 1-300bp downstream of the gene end. 3’ and 5’ UTR: Messenger RNA sequences that are untranslated and lie three prime/five prime to sequences which are translated.

**Supplementary Figure S4:**


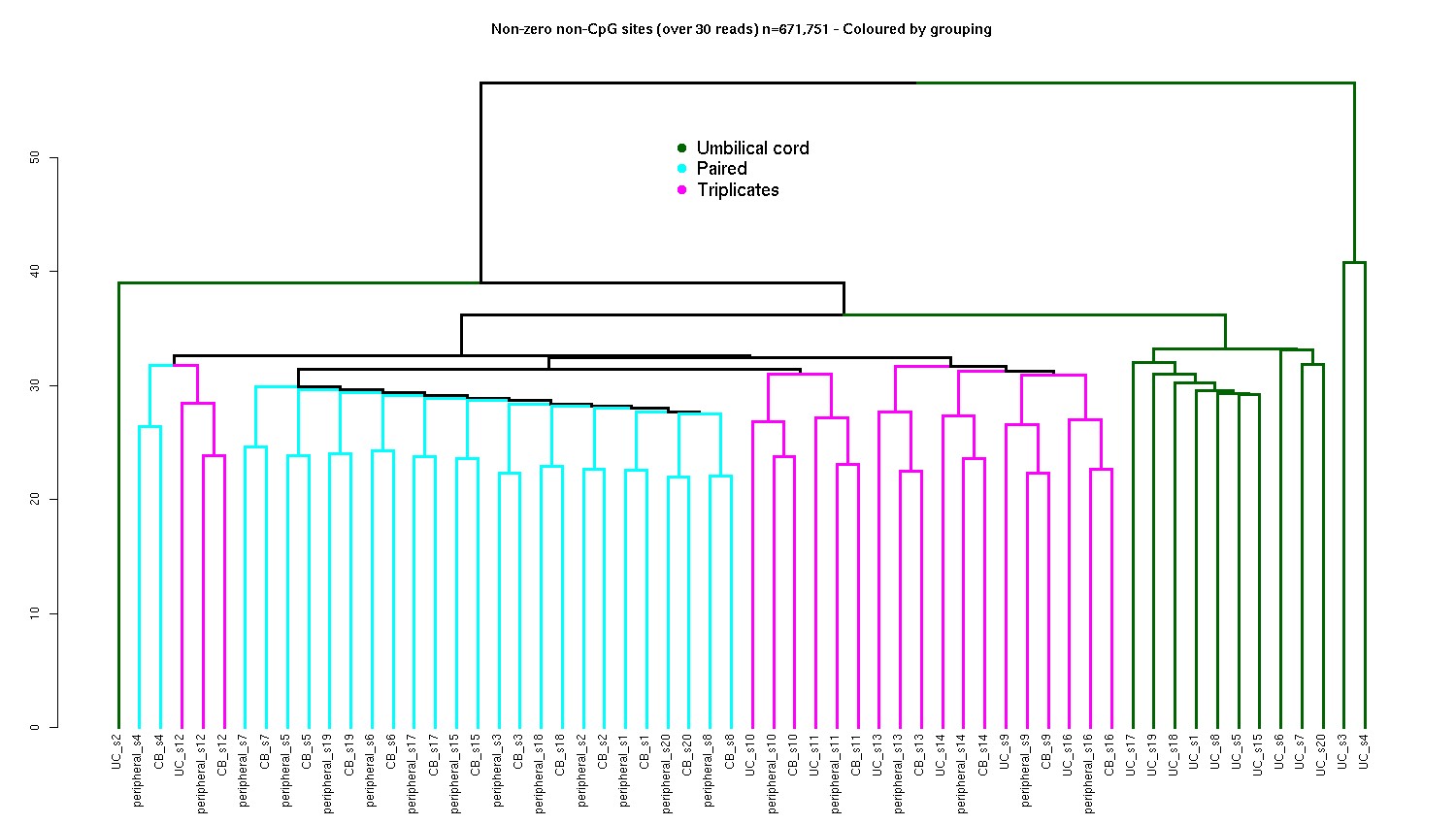


**Figure S4: Dendrogram of discovery dataset using 671,751 non-CpG sites:** these 671,751 non-CpG sites have >30x read-depth and non-zero methylation values across all 60 samples in discovery dataset. An individual’s peripheral blood and cord blood samples grouping in pairs (cyan), or triplicates of all three tissue types of an individual clustering together (magenta). CB= cord blood samples, UC= Umbilical cord samples (green), peripheral= peripheral blood samples.

**Supplementary Figure S5 (a - i):**

**Figure S5 (a-i): Dendrograms of samples using non-zero non-CpG sites by 3 base-pair genetic context**: Clustering of 60 samples from discovery dataset broken down by methylated cytosine environment (5’ to 3’). An individual’s peripheral blood and cord blood samples grouping in pairs (cyan), or triplicates of all three tissue types of an individual clustering together (magenta). Umbilical cord samples not in pairs or triplicates are shown in green, unpaired samples in black. Non-CpG 3 base-pair environments are: **(a)** CAG (n=141,517), **(b)** CAA (n=90,912), **(c)** CTA (n=16,560), **(d)** CTG (n=42,342), **(e)** CTT (n=31,542), **(f)** CCT (n=29,168), **(g)** CCA (n=37,993), **(h)** CCG (n=25,025), and **(i)** CCC (n=18,593).

**Supplementary Figure S6 (a – d):**

**Figure S6 (a–d):** **Dendrograms of samples using CpG sites split by three base pair sequence context:** Clustering of 60 samples from discovery dataset broken down by methylated cytosine environment (5’ to 3’). Cyan represents an individual’s peripheral blood and cord blood samples grouping in pairs, umbilical Cord samples are shown in green unpaired samples shown in black. CpGN 3 base-pair environments are: **(a) CGA** (n=246,003)**, (b) CGC** (n=325,115)**, (c) CGT** (n=275,546)**,** and **(d)** CGG (n=375,873).
